# Circulating exosomes with unique lipid signature in relapsing remitting multiple sclerosis

**DOI:** 10.3389/fncel.2025.1613618

**Published:** 2025-06-27

**Authors:** Claudia Palazzo, Ilaria Asci, Silvia Russo, Cinzia Buccoliero, Vincenzo Mangialardi, Pasqua Abbrescia, Onofrio Valente, Maddalena Ruggieri, Damiano Paolicelli, Simona Lobasso, Antonio Frigeri

**Affiliations:** ^1^Department of Translational Biomedicine and Neuroscience, University of Bari Aldo Moro, Bari, Italy; ^2^Department of Biosciences, Biotechnologies and Environment, University of Bari Aldo Moro, Bari, Italy

**Keywords:** extracellular vesicles, serum, lipid biomarkers, autoimmune disorders, MALDI-TOF/MS

## Abstract

Exosomes are small, membrane-bound vesicles secreted by most cell types into the extracellular environment. They play a crucial role in intercellular communication by transporting bioactive molecules, including proteins, lipids, and RNAs, thereby influencing the phenotype and potentially the genotype in recipient cells. In recent years, exosomes have gained increasing attention in the study of pathophysiological conditions and numerous diseases, including multiple sclerosis (MS), an autoimmune disorder with myelin sheath and neuroaxonal damage in the central nervous system. In this study, we isolated and purified serum-derived exosomes from patients with relapsing remitting MS (RR-MS) and characterized their lipid profiles using matrix-assisted laser desorption ionization-time-of-flight/mass spectrometry (MALDI-TOF/MS). Lipid analysis was performed in both negative and positive ion modes on intact exosomes, bypassing lipid extraction steps and significantly reducing sample-processing time. The lipid profiles of RR-MS exosomes were compared to those of exosomes isolated from the serum of healthy subjects (HS), and statistical analysis was applied to mass spectra to identify potential lipid biomarkers. The specific phospholipid marker of exosomal membranes, bis(monoacylglycero)phosphate (BMP), was clearly detected in both MALDI lipid profiles, with no significant differences in its content between the two sample groups. However, RR-MS exosomes exhibited significantly lower levels of phosphatidic acid (PA) compared to HS exosomes, despite PA being a key structural component of extracellular vesicles. Notably, comparative analysis revealed an enrichment of several lysophosphatidylcholine (LPC) species in RR-MS exosome membranes, aligning with their known proinflammatory role in MS pathology. Our most significant finding was a markedly lower phosphatidylcholine (PC) to LPC ratio in the pathological group indicating potential alterations in membrane lipid homeostasis. To the best of our knowledge, this study is the first to report a distinct lipid signature in serum-derived exosomes from RR-MS patients using direct MALDI-TOF/MS analysis.

## 1 Introduction

Multiple sclerosis (MS) is the most common chronic inflammatory, demyelinating, and neurodegenerative disease of the central nervous system (CNS), which primarily affects individuals between 20 and 40 years of age (Reich et al., 2018). MS is a heterogeneous, multifactorial, immune-mediated disease influenced by both genetic and environmental factors (Filippi et al., 2018), in which the immune system attacks myelin components, leading to axonal degeneration in the brain and spinal cord of MS patients. While MS has long been considered an autoreactive T-cell-driven pathology (Dendrou et al., 2015), more recent evidence indicates that the disease involves complex interactions among multiple lymphocyte subsets, including B cells (Li et al., 2018; Paolicelli et al., 2022). The clonal expansion of immunoglobulin-secreting B cells and plasma cells in the CNS results in the characteristic presence of cerebrospinal fluid (CSF)-specific oligoclonal bands (Housley et al., 2015). Although the precise targets of these immunoglobulins remain unclear, their presence signifies an intrathecal immune response.

Currently, MS diagnosis primarily relies on clinical and radiological findings, including neurological examination, magnetic resonance imaging, and CSF analysis for oligoclonal bands and kappa free light chains index (Reich et al., 2018; Alves Martins et al., 2022). To date, no validated blood-based molecular markers have demonstrated sufficient sensitivity and specificity for MS diagnosis.

Extracellular vesicles (EVs), particularly exosomes, have gained increasing attention as potential biomarkers and therapeutic agents in various diseases, including neurological disorders such as MS. As membrane-bound vesicles released by most cell types into the extracellular space, exosomes cross the blood-brain barrier (BBB) and their ability makes them promising candidates for CNS disease biomarker discovery.

A pivotal study in 1989 first reported the presence of oligodendroglial vesicles in the CSF of MS patients paving the way for research into their role in MS (Scolding et al., 1989). Then, CSF-derived exosomes have been found to carry specific miRNAs that are associated with MS progression and severity (Wang and Zhang, 2020). Today, exosomes are well-recognized for their ability to activate immune cells and modulate the inflammatory response within the CNS in MS (Hasaniani et al., 2024).

Recent advancements in high-resolution mass spectrometry and lipidomic workflows have enabled comprehensive profiling of exosomal lipids, shedding light on their roles in various diseases. For instance, a recent study identified a novel diagnostic marker for type 2 diabetes mellitus (T2DM) by analyzing lipid metabolites in serum exosomes. The study revealed that phosphatidylethanolamine (PE 36:2) levels were elevated in T2DM serum exosomes and correlated with hemoglobin A1C and blood glucose levels (Zhang et al., 2024).

Various serum exosomal lipids also resulted differentially expressed between schizophrenia patients and healthy controls, revealing dysregulated lipid metabolism pathways. Among these, specific ceramides and PE suggested their integral role in the disorder’s pathophysiology (Xu et al., 2025).

In the context of MS, earlier studies have highlighted lipids as key mediators of autoimmune brain inflammation and have identified unique lipid antibody patterns corresponding to different pathological stages of the disease (Kanter et al., 2006; Quintana et al., 2008). Recent works have significantly advanced our understanding of lipid metabolism alterations in relapsing-remitting multiple sclerosis (RR-MS), highlighting potential biomarkers and therapeutic targets. A 2024 study utilizing high-resolution mass spectrometry analyzed plasma samples from RR-MS patients, chronic progressive MS patients, and healthy controls. Notably, RR-MS patients exhibited increased levels of diacylglycerols (DAGs), which showed high predictive value for MS. Distinct lipidomic signatures were found, such as alterations in PEs mainly linked to RR-MS, while ether-bound PEs were associated with chronic progressive MS (Lattau et al., 2024).

Using LC-MS/MS-based targeted lipid mediator profiling, some researchers observed distinct changes in omega fatty acid-derived metabolites in RR-MS patients compared to healthy subjects. Specifically, certain pro-inflammatory mediators were elevated, while other lipid mediators were decreased in patients. This imbalance between pro-inflammatory and pro-resolving lipid mediators may contribute to the chronic inflammation characteristic of MS (Zahoor et al., 2024).

Moreover, emerging research indicates that dysregulated lipid metabolism can modulate T-cell function in people with RR-MS. Alterations in lipid metabolism networks may influence immune cell signaling and function, potentially contributing to MS pathogenesis (Martin-Gutierrez et al., 2024).

Understanding these mechanisms could lead to novel therapeutic approaches targeting lipid metabolism pathways.

In this context of exosomal lipid profiling in MS, our study represents the first MALDI-TOF-based lipidomic investigation of serum exosomes from RR-MS patients, with the aim of characterizing their lipid composition and identifying novel potential biomarkers.

## 2 Materials and methods

### 2.1 Subjects

We recruited 10 RR-MS patients with ages spanning from 26 to 60 years old. They were recruited at the Neurology Unit of University of Bari Aldo Moro (Italy) and underwent blood sampling. Among the enrolled RR-MS patients, with the mean age of 40.7 ± 11.4 years, 3 patients were treatment naïve. The demographic and clinical characteristics of the patients are reported in the following Table 1.

**TABLE 1:** Demographic and clinical characteristics of the RR-MS patients.

Characteristics	Entire cohort (*n* = 10)
Age, mean years ± SD (range)	40.7 ± 11.4 (26.0–60.0)
Disease duration, mean years ± SD (range)	4.90 ± 6.90 (0.06–22.03)
Women, *n* (%)	6 (60%)
EDSS, median (IQR)	2.5 (1.5–5.0)
Previous therapy, *n* (%):	
First line	5 (50%)
Second line	2 (20%)
Naïve	3 (30%)
Previous treatment duration, mean years ± SD	3.4 ± 2.9

EDSS, Expanded Disability Status Scale; IQR, interquartile.

An additional group of 8 serum samples from age-matched healthy subjects (HS) were also collected. The study design was conducted following the guidelines for the local Ethics Committee that approved the study (Study REGISTRO SM001 – approved on 8 July 2016 by the Ethic committee of Policlinico of Bari, Italy), and conducted according to Declaration of Helsinki (World Medical Association, 1997). All subjects were informed about the procedures and provided written informed consent to participate in the study. To protect human subject identity a code number was employed for specimen identification. All patients were selected to obtain cohorts matched by age, sex and ethnicity (Caucasian individuals). Patients with other MS disease courses and relevant comorbidities were excluded. All serum samples were collected at the Neurology Unit of University of Bari (Italy). Samples underwent stringent quality control checks prior to inclusion in the study to ensure consistency in pre-analytical variables, such as hemolysis, storage conditions, and freeze-thaw cycles.

### 2.2 Exosomes isolation

Whole blood samples (∼9 mL) were collected from HS and RR-MS patients.

Samples with limited volume were excluded. First cellular components were removed by centrifugation (3,000 × *g*, 4°C, 7 min). Then two procedures were used for serum-derived exosomes isolation.

For the commercially available isolation assay, each sample was purified using the ExoQuick ULTRA EV Isolation Kit for Serum and Plasma (SBI-System Biosciences, Palo Alto, CA, United States), following the manufacturer’s protocol.

For the ultracentrifugation isolation method, blood sera (∼5 mL) were diluted 1:1 with phosphate-buffered saline (PBS, Gibco, Hampton, NH, United States), and centrifuged at 2,000 × *g* at 4°C for 30 min.

Supernatants were then filtered through a 0.8 μm filter (Millipore, Burlington, Massachusetts, United States) by hydrostatic pressure to remove remaining platelets and apoptotic bodies and then centrifuged at 12,000 × *g*, 4°C for 45 min to remove microvesicles. Supernatants were then filtered through 0.22 μm filters (Millipore, Burlington, Massachusetts, United States) and then again centrifuged at 120,000 × *g*, 4°C, for 70 min in an ultra-high speed centrifuge. This procedure was repeated twice. The supernatants were then carefully discarded. Finally the small pellet at the bottom of the centrifuge tube, containing exosomes, was resuspended in ∼ 50 μL PBS containing complete protease inhibitors cocktail (Roche, Milan, Italy) and stored at −80°C.

### 2.3 Exosomes characterization

After isolation, the characterization of exosomes obtained with both the techniques described above has been performed by measuring the expression of tetraspanins TSG101, CD63 and CD81. SDS-PAGE and Western blot have been performed as previously described (De Bellis et al., 2017; Palazzo et al., 2020). Briefly, proteins dissolved in Laemmli sample buffer were resolved on a 13% polyacrylamide gel, and transferred onto PVDF membranes (Immobilon PVDF; Millipore, Burlington, Massachusetts, United States) for immunoblot analysis. After transfer, membranes were blocked and incubated with primary antibodies as described in the next “Antibodies” section. After washing, membranes were incubated with peroxidase-conjugated secondary antibodies and washed again. Reactive proteins were revealed with an enhanced chemiluminescent detection system (Clarity Western ECL substrate, Bio-Rad, Hercules, CA, United States) and visualized on a ChemiDoc Touch imaging system (Bio-Rad, Hercules, CA, United States). Images were analyzed using Image Lab (Bio-Rad, Hercules, CA, United States). Rouge Ponceau staining was used to verify total protein loading across the lanes and to correct (normalize) the protein content in each lane.

Exosomes dimension has been determined through Dynamic Light Scattering (DLS) method by a Malvern Zetasizer Lab ZS (Malvern Panalytical, Great Malvern, United Kingdom). The size of the exosomes has been calculated from the translational diffusion coefficient by using the Stokes-Einstein equation.

### 2.4 Antibodies

The following primary antibodies were used for immunoblot analysis: Rabbit Recombinant Monoclonal anti-TSG101 antibody (Abcam, Cambridge, United Kingdom) diluted to 1:1000, Mouse Monoclonal anti-CD63 antibody (Abcam, Cambridge, United Kingdom) diluted to 1:1000, Rabbit polyclonal anti-CD81 antibody (SBI-System Biosciences, Palo Alto, CA, United States) diluted to 1:1000; as secondary antibody, anti-rabbit IgG-HRP was used, following the manufacturer’s instructions (Bio-Rad, CA, United States).

### 2.5 Lipid analysis by MALDI-TOF/MS

Matrix-assisted laser desorption ionization-time-of-flight mass spectra of isolated exosomes were acquired on a Bruker Microflex LRF mass spectrometer (Bruker Daltonics, Bremen, Germany). The matrix 9-aminoacridine hemihydrate (9-AA) was purchased from Acros Organics (Morris Plains, NJ, United States). All mass spectra were acquired in both negative and positive ion mode in reflector mode (detection range: 200–2,000 mass/charge, *m/z*) using the delayed pulsed extraction. For each mass spectrum, 2,000 single laser shots were averaged. The laser fluence was about 5% above threshold to have a good signal-to-noise (S/N) ratio. Peak areas, baseline correction, spectral mass resolutions and S/N ratios were determined by *Flex Analysis 3.3* software (Bruker Daltonics, Bremen, Germany). The commercial lipids (used as external standards) were purchased from Avanti Polar Lipids (Alabaster, AL, United States). External calibration with lipid standards was performed before each measurement, as previously described (Lobasso et al., 2017; Lopalco et al., 2019). Lipids of exosomes were directly analyzed by the “intact method” (Angelini et al., 2012; Russo et al., 2022). In brief, the isolated exosomes were diluted in H_2_O to 1.0 μg/μL of total protein concentration, determined by Bradford method. Then 1 μL of suspension was spotted on the MALDI target (Micro Scout Plate, MSP 96 ground steel target). After H_2_O evaporation, a thin layer (0.35 μL) of matrix solution (20 mg/mL 9-AA in 2-propanol/acetonitrile, 60/40, by vol.) is spotted on the dried sample. Finally, even after matrix evaporation, MALDI-TOF/MS analysis of exosomal lipids was performed.

Series of MALDI mass spectra (three replicates for each sample) were averaged using the software for the mass spectrometer *ClinProTools* 3.0 (Bruker Daltonics, Bremen, Germany) to find the area under the peaks. Statistically significant differences between series of mass spectra obtained from RR-MS and HS exosomes were determined using Student’s *t*-test when dependent variable was normally distributed and Wilcoxon/Kruskal-Wallis test when the distribution did not fit the normally distribution. A *p*-value < 0.05 was set as a threshold statistically significant differences.

A specific on-line database resource (Lipid Maps Database)^1^ was used for lipid identification.

## 3 Results

### 3.1 Exosomes isolation and characterization

**FIGURE 1:**
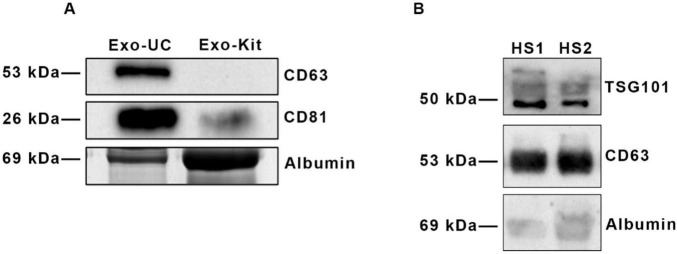
Comparison of surface marker profiling of exosomes isolated by two different techniques. **(A)** Immunoblot analysis of exosomes isolated by the ultracentrifugation protocol (Exo-UC) and the commercial kit (Exo-Kit), probed with anti-CD63 and anti-CD81 antibodies. Albumin contamination (∼69 kDa) is also shown. **(B)** Immunoblot analysis of two different samples of exosomes isolated from HS sera (HS1, HS2), showing reproducible enrichment of the surface markers TSG101 and CD63, with minimal albumin contamination.

Experiments were performed to set up the optimal method and the proper amount of peripheral blood required for the isolation of exosomes from serum. In this study, we took advantage of two different isolation techniques: differential ultracentrifugation (Exo-UC) and a commercial kit (Exo-Kit), as described in Method section. After isolation, their characterization was performed to evaluate the presence of tetraspanins, as specific exosomal surface markers. Therefore, exosomes isolated from HS serum samples were analyzed by SDS-PAGE analysis, followed by immunoblot for CD81, CD63 and TSG101 (Figure 1). Exosomes isolated by differential ultracentrifugation showed a higher enrichment of specific surface protein markers compared to those isolated by the commercial kit. As shown in Figure 1A two intense bands are visible in Exo-UC at ∼26 kDa and ∼53 kDa, corresponding to CD81 and CD63, respectively, while the Exo-Kit sample displays only a faint band corresponding to CD81. Furthermore, a significant albumin contamination was detected in the preparation obtained from the Exo-Kit (Figure 1A).

In addition, Figure 1B shows representative results from two different HS exosomes preparations (HS1 and HS2). Both samples displayed two prominent bands at the expected size of ∼50 kDa and ∼53 kDa, corresponding to the tetraspanins TSG101 and CD63, respectively, with a minimal albumin contamination (Figure 1B). These data highlight the reproducibility of the ultracentrifugation method, in terms of exosomes enrichment and exosomal protein marker abundance. Similar results were obtained when exosomes isolated from RR-MS patients were analyzed (not shown).

These data indicate that Exo-UC is the most adequate technique for exosomes isolation. In fact, our analysis revealed that the Exo-UC method consistently provided higher particle-to-protein ratios, indicative of greater purity and lower albumin contamination. Moreover, this method produced more robust and specific exosomal marker signals in immunoblotting, supporting its use for downstream lipidomic profiling throughout our study.

**FIGURE 2:**
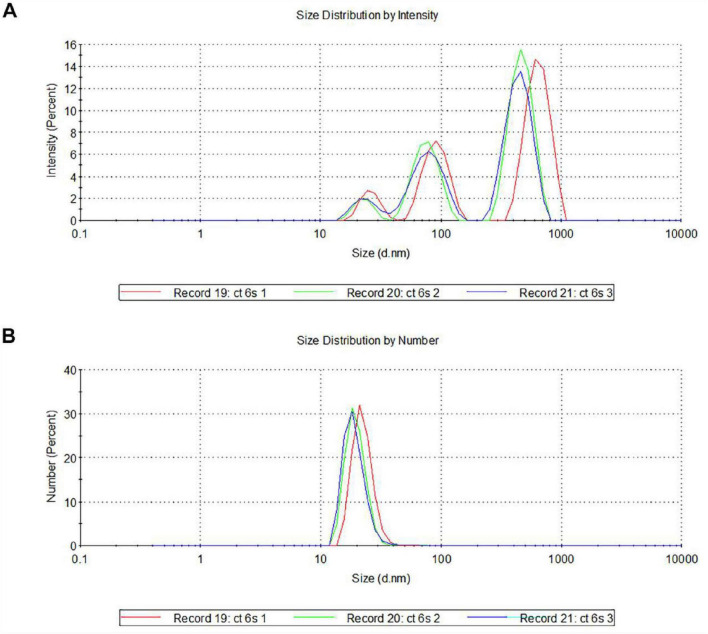
Characterization of serum-derived exosomes by DLS. Representative hydrodynamic radius distributions of exosomes measured by intensity distribution **(A)** and by number **(B)**. Note that the spectra, performed in triplicate (named as record 19, 20, and 21), indicate the presence of vesicular structures at the predicted size of 30–100 nm **(A,B)**.

Then, we used Dynamic Light Scattering (DLS) to measure the diameter of isolated vesicles, confirming that they fall within the typical exosomal range (30–100 nm). To characterize exosomes derived from serum, the suspensions were subjected to three runs (3 min each) with a scattering angle of 90° and intensity-weighted and number distributions were calculated (Figure 2).

The size distribution of exosomes revealed an evident bimodality when analyzed by their intensity, with two distinct major peaks in the 80–100 nm and 800–1,000 nm size ranges (Figure 2A). Peaks in the smaller sizes range could correspond to protein aggregates or microsomes, while peaks around 1,000 nm could represent larger particles, such as microvesicles or exosome aggregates. Looking at the particle number distributions, a predominant population of particles in the 30–50 nm size range emerges (Figure 2B).

Thus, the combined use of immunoblotting and DLS analyses provided clear evidence of the presence and integrity of the isolated serum-derived exosomes.

### 3.2 MALDI comparative lipidomic analysis of HS and RR-MS exosomes

Isolated exosomes were analyzed by MALDI-TOF/MS using “intact” method (Angelini et al., 2012) to identify potential changes in their lipid components.

**FIGURE 3:**
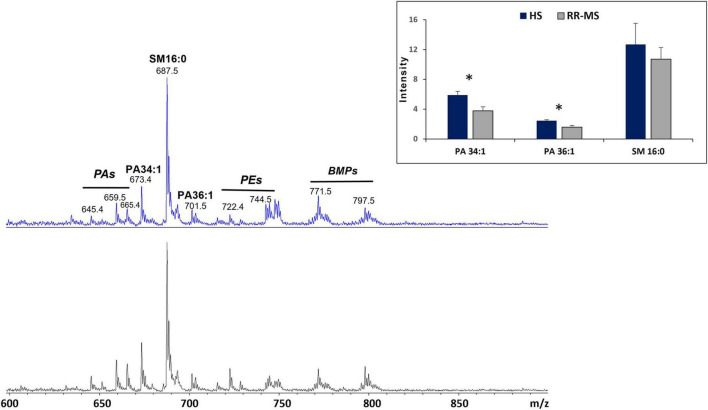
(−) MALDI-TOF/MS lipid profiles of exosomes obtained from HS (upper panel) and RR-MS patients (lower panel). Representative mass spectra obtained in negative ion mode are showed and lipid assignments for main MALDI *m/z* peaks are reported. The histograms (in the inset) show the differences in intensity between some lipid peaks present in the two series of (−) mass spectra, with *p*-value < 0.05 (*) set as the threshold to define significant differences. Data are reported as the average value of intensity ± SD. PA, phosphatidic acid; PE, phosphatidylethanolamine; SM, sphingomyelin; BMP, bis (monoacylglycero) phosphate.

Figure 3 compares the representative lipid profiles of serum-derived exosomes isolated from HS and RR-MS patients, obtained by negative ion mode MALDI-TOF/MS analysis. The main signals in the MALDI-TOF mass spectra, corresponding to the negative [M-H]^–^ molecular ions of main detected lipid classes, are summarized in Table 2.

**TABLE 2:** Lipid assignments of *m/z* values detected in negative and positive ion mode MALDI-TOF, mass spectra of isolated serum-exosomes.

*m/z* value	[M-H]^–^	[M + H]^+^
496.5	–	LPC (16:0)
518.5	–	LPC (18:3)
524.4	–	LPC (18:0)
546.5	–	LPC (20:3)
550.5	–	LPC (20:1)
645.4	PA (32:1)	–
659.5	PlsA (P-34:0)/PlsA (O-34:1)	–
665.5	PA (34:5)	–
673.4	PA (34:1)	–
687.5	SM (16:0)	–
695.4	PA (36:4)	–
701.5	PA (36:1)	–
703.5	–	SM (16:0)
722.4	PE (P-36:4)	–
727.5	–	SM (18:2)
742.4	PE (36:2)	–
744.5	PE (36:1)	–
758.5	–	PC (34:2)
760.5	–	PC (34:1)
771.5	BMP (36:3)	–
782.5	–	PC (36:4)
784.5	–	PC (36:3)
797.5	BMP (38:4)	–
799.6	BMP (38:3)	–
810.5	–	PC (38:4)

The numbers (x:y) denote the total carbon numbers and number of double bonds in acyl chains, respectively. Pls, plasmalogen.

The two lipid profiles showed in Figure 3 exhibit many similarities, with the main peaks corresponding to major glycerophospholipid and sphingolipid species (*m/z* range 600–950). The peak at *m/z* 687.5, which corresponds to the sphingophospholipid sphingomyelin (SM 16:0) containing a palmitic acid, dominates both lipid patterns. The signals at *m/z* 645.4, 659.5, 673.4, and 701.5 are assigned to various phosphatidic acid (PA) species. Additionally, the smaller peaks at *m/z* 722.4, 742.4, and 744.5 may be attributable to PE species (see Table 2).

Additional MALDI signals are visible at *m/z* 771.5, 797.5, and 799.6, which are assigned to various species of a distinctive polyglycerophospholipid, known as bis(monoacylglycero)phosphate (BMP) (Figure 3). The peculiarity of its chemical structure lies in the presence of two glycerol molecules with the two acyl-chains that are esterified at the *sn*-1 position of each glycerol. In contrast, in phosphatidylglycerol (PG) structure the two fatty acids are esterified at the same glycerol backbone (Holbrook et al., 1992; Hankin et al., 2015). The detection of BMP in our exosomes is noteworthy as it is considered a novel, specific endolysosomal lipid marker, potentially indicating the endosomal origin of small EVs (Hullin-Matsuda et al., 2022).

No other major peaks are visible in higher *m/z* range of mass spectra of exosomal lipids, where complex glycosphingolipids and the phospholipid cardiolipin are typically detected (not shown).

By comparing series of replicates of (-) mass spectra exosomes, we found that two signals at *m/z* 673.7 and 701.5, attributable to PA 34:1 and PA 36:1, respectively, were significantly lower in RR-MS samples than in HS samples (Figure 3, inset). This suggests that PA species, containing one saturated and one monounsaturated acyl chain, are less abundant in RR-MS exosomes. No other significant differences were observed in the MALDI negative ion mode analyses between the two samples, including SM 16:0 content (peak at *m/z* 687.6) that was similar, as shown in Figure 3, inset.

**FIGURE 4:**
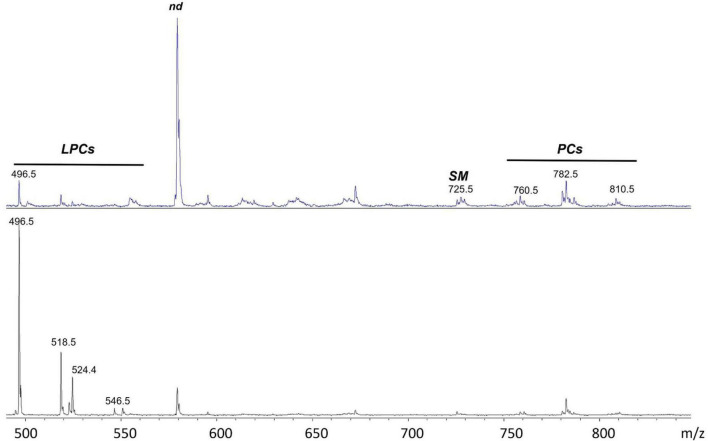
(+) MALDI-TOF/MS lipid profiles of exosomes obtained from HS (upper panel) and RR-MS patients (lower panel). Representative mass spectra obtained in positive ion mode are showed and lipid assignments for main MALDI *m/z* peaks are reported. LPC, lysophosphatidylcholine; SM, sphingomyelin; PC, phosphatidylcholine; nd, peak of no interest.

Figure 4 compares the representative lipid profiles of HS and RR-MS exosomes, obtained by positive ion MALDI-TOF/MS analyses. The main signals in the (+) mass spectra, corresponding to the [M + H]^+^ molecular ions of glycerophospholipids and sphingophospholipids, are summarized in Table 2.

Disregarding the higher peak (at *m/z* 579.3) in the upper mass spectrum that is not of our interest, probably due to an excess of matrix, it is clear that the two lipid patterns are different, especially for the peaks that are detectable in the lower 450–550 *m/z* range. MALDI signals at *m/z* 496.7, 518.7, 524.4, 546.5 and 550.5 are present in both the exosomal mass spectra (Figure 4) and are assigned to the [M + H]^+^ molecular ion of the lysophosphatidylcholine (LPC) species. In particular, the signal at *m/z* 496.7, attributed to the lysophospholipid LPC with a palmitic acid (LPC 16:0), is dominant in the lipid pattern of RR-MS exosomes (Figure 4, lower panel).

Smaller signals in the higher *m/z* range, at *m/z* 760.5, 782.6 and 810.5, attributable to the [M + H]^+^ molecular ions of three phosphatidylcholine (PC) species, are detectable in both mass spectra, with no evident differences in intensity.

Furthermore, the molecular species SM 16:0 was found in both exosome lipid profiles as low signals at *m/z* 703.5 and 725.5, corresponding to the molecular ion and the sodiated form of the same lipid, respectively. This finding is consistent with its presence previously described using (-) MALDI analysis where it appeared as an intense peak at *m/z* 687.5 (see Figure 3).

**FIGURE 5:**
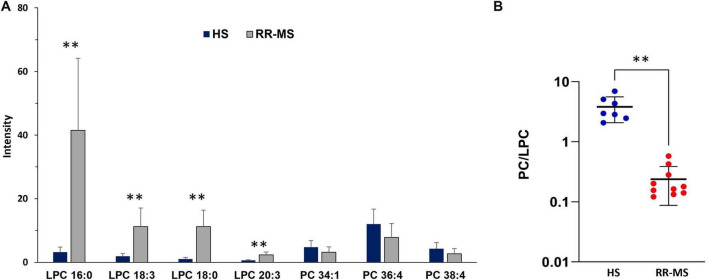
Differences in PC and LPC contents **(A)** and PC/LPC ratio **(B)** between HS and RR-MS exosomes. The histograms **(A)** illustrate the differences in both LPC and PC signals detected in the two series of (+) MALDI mass spectra, with *p*-value < 0.05 set as the threshold to define significant differences. Data are reported as the average value of intensity ± SD (**p < 0.01). The plot **(B)** illustrates the calculated PC/LPC ratios in exosomes obtained from HS (blue dots) and RR-MS (red dots) (**p < 0.01). LPC, lysophosphatidylcholine; PC, phosphatidylcholine.

The histograms in Figure 5A show the results of the statistical analysis of peak intensities between HS and RR-MS, as detected in the MALDI positive ion mass spectra of exosomes. The signals at *m/z* 496.7, 518.7, 524.4 and 546.5, assigned to the [M + H]^+^ molecular ions of the LPC 16:0, 18:3, 18:0 and 20:3 species, respectively, were significantly higher in the lipid profile of RR-MS exosomes than in HS ones.

Regarding the PC species, the peaks at *m/z* 760.5, 782.6 and 810.5, attributable to PC 34:1, 36:4, and 38:4 species, respectively, showed a tendency toward lower intensities in the lipid profiles of RR-MS exosomes compared to HS exosomes, but the differences were not statistically significant (Figure 5A). Figure 5B shows that the PC/LPC ratios calculated from the various MALDI mass spectra of RR-MS exosomes were significantly lower than those of HS exosomes.

These findings reveal a distinct lipid signature in circulating exosomes from RR-MS patients, characterized by a significant lower PA content and higher LPC level compared to HS exosomes. Notably, the PC/LPC ratio, a lipid marker generally associated with inflammatory states (Fuchs et al., 2005; Angelini et al., 2014), was significantly decreased in lipid profiles of RR-MS exosomes due to LPC enrichment in their membranes.

## 4 Discussion

Exosomes derived from both normal and cancerous cells can be widely found in various body fluids such as plasma, saliva, urine and CSF. Although they were previously considered as mediators of cellular debris degradation, more recent studies have demonstrated that these nano-sized vesicles, carrying nucleic acids, lipids, proteins, enzymes and miRNAs, play crucial roles in intracellular trafficking and pathophysiological states.

A major challenge in EV research is the lack of standardized methodologies for assessing their downstream applicability, purity, and yield from biological fluids such as serum. Several exosome isolation techniques exist, yet they often suffer from limitations such as low recovery rates, poor reproducibility, and the requirement for costly reagents and equipment (Lobb et al., 2015; Alexander et al., 2016). Among these, differential centrifugation remains the most widely used method for exosome isolation (Lobb et al., 2015).

In this study, we compared two different exosome isolation techniques—differential ultracentrifugation and a commercial one-step isolation kit—to determine the most appropriate method based on surface protein analysis and DLS for size characterization. Here, we demonstrate that differential ultracentrifugation provides higher particle purity with minimal albumin contamination and is well suited for downstream applications, particularly lipidomic analysis.

The lipid composition of EV/exosomes has received relatively less attention in the scientific literature compared to proteomic analyses. While the number of studies on exosomal lipid profiling is increasing in PubMed, it remains lower than those focusing on proteins. Lipidomics studies have demonstrated that the lipid profile of EVs varies depending on their cellular origin, although it differs from that of their parent cells reflecting selective packaging processes during their biogenesis (Record et al., 2014; Skotland et al., 2019; Lobasso et al., 2021). For example, exosomal membranes are known to be enriched in specific lipid species, including cholesterol, PC, and SM, which influence exosomal stability, curvature, and interaction with recipient cells (Ghadami and Dellinger, 2023).

Exosomal lipidomics is a rapidly evolving field that offers valuable insights into the lipid composition of exosomes. Multiple recent studies have highlighted the crucial role of exosomal lipid composition in their mechanism of action, emphasizing that shifts from normal physiological conditions to pathophysiological states can alter their lipid profile (Ghadami and Dellinger, 2023). Notably, the involvement of exosomes in various CNS disorders is well established. In particular, exosomes play a key role in the early stages of MS (Mohammadinasr et al., 2024).

To our knowledge, this is the first MALDI-based study characterizing the lipid signature of circulating exosomes isolated from RR-MS patients. Here, we employed direct MALDI-TOF/MS analysis — an extremely sensitive analytical technique — to analyze comprehensively the lipid profile of serum-derived RR-MS exosomes in comparison to those isolated from healthy controls. This method enables the analysis of minute amounts of biological material while bypassing lipid extraction and reducing sample processing time (Angelini et al., 2012). Specifically, only 1 μL of aqueous exosome suspension (at a protein concentration of 1 μg/μl) was loaded onto the MALDI target to obtain a detailed lipid profile. MALDI mass spectra of exosomes were acquired in both (−) and (+) ionization modes using the same matrix (9-AA) to detect key lipid species. Acidic lipid classes were preferentially ionized in (−) ion mode, while zwitterionic phospholipids were better detected in (+) ion mode, depending on their chemical structures.

MALDI-TOF/MS analysis in (−) ion mode revealed similar lipid profiles for RR-MS and HS exosomes, including the major sphingophospholipid species SM16:0 and various species of the glycerophospholipid PE. Comparative mass spectra analysis showed no significant differences in signal intensity between the two groups of exosomes.

Interestingly, both types of exosomes exhibited the presence of the polyglycerophospholipid BMP, an unusual molecule containing two monoacylglycerol moieties linked through a phosphate group (Holbrook et al., 1992). BMP is recognized as a specific endolysosomal lipid marker, indicating the endosomal origin of small EVs, known as exosomes (Hullin-Matsuda et al., 2022). A recent lipidomic study of exosomes from melanoma cells reported the presence of various BMP species in their membranes and demonstrated its enrichment in exosomal membranes compared to their parental cells (Lobasso et al., 2021).

While we found no significant differences in BMP content between control and RR-MS exosomal profiles, to our knowledge, no previous studies described its presence in serum-derived exosomes from RR-MS patients. On other hand, the role of BMP in endolysosomal function suggests several potential implications, such as inflammatory signaling and biomarker potential in RR-MS patients. Exosomal BMP might participate in modulating immune responses, potentially influencing the inflammatory environment characteristic of the disease. Given its association with endolysosomal compartments, BMP in exosomes could serve as a biomarker for monitoring disease progression or response to therapy.

Further research is needed to elucidate the specific roles of BMP-containing exosomes in RR-MS and their potential as diagnostic or therapeutic targets.

Regarding the sphingophospholipid SM, it is crucial for myelin formation and undergoes degradation via the sphingomyelinase enzyme through the exocytosis pathway (Kornhuber et al., 2015). In MS, autoimmune attacks target myelin components, leading to axonal degeneration. A 2008 study on post-mortem brains of MS patients reported reduced sphingolipid levels compared to control tissues (Wheeler et al., 2008). Additionally, decreased SM levels have been observed in the CSF of individuals with MS, likely due to the action of sphingomyelinase-enriched exosomes (Pieragostino et al., 2018). However, in our study, comparative MALDI analyses of serum-derived exosomes revealed no significant differences in SM levels.

Notably, statistical analysis of the MALDI lipid profiles revealed that two PA species, both containing one saturated and one monounsaturated fatty acid, were significantly enriched in RR-MS exosomes compared to controls. PA, characterized by a small headgroup, is a cone-shaped phospholipid that induces negative membrane curvature (Tanguy et al., 2019). It is considered one of the main lipid types in EVs and plays a key role in the formation of intraluminal vesicles as well as in regulating the number of EVs released from cells (Ghadami and Dellinger, 2023). A MALDI-TOF untargeted lipidomics analysis of CSF from MS patients previously reported significantly reduced levels of PA34:1 compared to individuals with other neurological diseases (Pieragostino et al., 2015), in agreement with our findings.

Comparative MALDI-TOF analysis in (+) ion mode revealed striking differences in serum-derived exosomes: a significantly increased content of LPC species in RR-MS exosomes compared to controls. In particular, LPC species with saturated acyl chains (palmitic or stearic acid) and with polyunsaturated fatty acids (PUFAs) (such as C18:3 and C20:3) were enriched in RR-MS exosomes, with the unsaturated LPC 16:0 species emerging as the dominant signal. Alterations in acyl composition, particularly an increase in saturated fatty acid tails in the main LPC species, may affect the physical properties of RR-MS exosomal membranes by reducing membrane fluidity and influencing lipid domains and lipid-protein interactions.

In general, LPC is primarily derived from the turnover of PC, the major phospholipid component of the cell membrane, through cleavage by phospholipase A2 (PLA2) (Liu et al., 2020). Autotaxin, a lysophospholipase D, can subsequently convert LPC into lysophosphatidic acid (LPA) (Moolenaar and Perrakis, 2011). However, in our study, no MALDI signals corresponding to LPA were detected in (-) ion mode mass spectra of RR-MS exosomes. This contrasts with previous reports describing increased autotaxin activity and elevated LPA levels in MS patients’ serum (Balood et al., 2014; Zahednasab et al., 2014).

In the literature, increased LPC content has been reported in diabetes, cardiovascular diseases, atherosclerosis, cervical cancer and inflammatory conditions (Liu et al., 2020). These lysocompounds are positively associated with neurodegenerative diseases, particularly with MS due to their proinflammatory properties (Zahednasab et al., 2014). In this context, it is known that LPC activates toll-like receptors (TLRs), especially TLR2 and TLR4, and this activation leads to the stimulation of macrophages and microglia, resulting in the release of pro-inflammatory cytokines such as interleukin (IL-1β) and tumor necrosis factor (TNF-α), thereby amplifying local CNS inflammation (Liu et al., 2020). Then LPC is a well-established demyelinating agent in animal experimental models, because its integration into myelin membranes leads to myelin breakdown and oligodendrocyte stress or death. LPC-induced demyelination is thought to occur through the actions of recruited macrophages and microglia, which phagocytose nearby myelin. Mechanisms of LPC-induced demyelination have been observed in MS, as LPCs stimulate the phagocytosis of the myelin sheath by macrophages in mice (Chu et al., 2019).

Therefore increased levels of LPC species in RR-MS might have several functional implications, particularly in the context of neuroinflammation and demyelination, which are central features of the pathophysiology.

Moreover elevated LPC levels are often associated with BBB dysfunction, allowing more immune cells and serum factors to infiltrate the CNS, and with oxidative stress and lipid peroxidation, further damaging CNS tissue (Chaves et al., 2024).

According to our findings, the previously cited study by Pieragostino et al. (2015) reported an increased content of two LPC species (LPC18:0 and LPC 18:1) in MS patients and identified these lysocompounds as potential lipid biomarkers correlated with clinical data (Link index) (Pieragostino et al., 2015).

Using a simple method which was previously developed and validated with horse serum (Angelini et al., 2014), we detected for the first time a reduced PC/LPC ratio in RR-MS exosomes compared to the control group by direct MALDI analysis.

Extensive scientific literature describes alterations in the PC/LPC ratio across various diseases. The simple PC/LPC measurement is generally considered as non-specific biomarker for inflammatory states which can be used for monitoring disease stages as well as treatments response (Mulder et al., 2003; Fuchs et al., 2005; Angelini et al., 2014). Regarding MS patients, earlier studies on blood plasma and serum have yielded conflicting results. Older studies reported increased LPC levels in plasma from MS patients (Andreoli and Cazzullo, 1969; Andreoli et al., 1973), whereas recent papers have described an increased PC/LPC ratio in serum (Del Boccio et al., 2011).

The altered PC/LPC ratio in RR-MS patients underscores the role of lipid metabolism dysregulation in the disease’s pathophysiology. Functional implications of reduced PC/LPC ratio include enhanced PLA2 activity (i.e., increased conversion of PC to LPC), which is associated with heightened lipid turnover and inflammatory responses in the disease.

Thus, the PC/LPC ratio might be considered an indicator of both active neuroinflammatory processes in RR-MS, correlating with disease relapses and progression, and neuronal membrane integrity disruption, contributing to demyelination and neuronal damage. Monitoring this ratio could provide insights into disease activity and serve as a potential biomarker for diagnosis and therapeutic targeting.

As regards the heterogeneous cell populations involved in the production of circulating exosomes, various CNS resident cells, such as neurons, astrocytes, oligodendrocytes, and microglia, are capable of releasing exosomes in the bloodstream (Hamzah et al., 2021; Badhwar et al., 2024). The identification of the cellular origin of circulating exosomes is still an ongoing challenge in the field, particularly within complex pathologies such as MS.

Discriminating the source of exosomes could provide essential insights into disease mechanisms. For instance, whether exosomal cargo reflects neurodegenerative damage, glial activation, or peripheral immune activity may critically influence their utility as biomarkers or therapeutic targets (Lucien et al., 2023). However, the precise attribution of biofluid-derived exosomes to specific cellular sources remains difficult due to the heterogeneity of EV populations in the blood and the current lack of universally accepted cell-type-specific markers (Park et al., 2024; Welsh et al., 2024).

Emerging technologies, including nano-flow cytometry and immunoaffinity-based EV enrichment, offer promising strategies for tracing exosome origin with greater specificity (Welsh et al., 2024; Yim et al., 2023). Integration of these approaches in future comparative studies across MS subtypes is necessary to better understand whether distinct exosomal lipidomic signatures correspond to specific disease mechanisms or cell types.

In conclusion, our findings contribute to a more comprehensive understanding of the lipid composition of exosomes derived from the serum of RR-MS patients. However, the limited sample size may have influenced our experimental results, and further investigation is needed to refine our lipid analysis.

More detailed and comprehensive lipidomic studies are required to better characterize the complex pathophysiological dynamics of exosomes, ultimately guiding their engineering for therapeutic applications and disease diagnostics. As research progresses, the integration of lipidomic analyses with other omics approaches will provide a more comprehensive understanding of MS mechanisms and facilitate the development of personalized medicine.

## Data Availability

The raw data supporting the conclusions of this article will be made available by the authors, without undue reservation.

## Footnotes

1
http://www.lipidmaps.org/tools
